# Oligocene and early Miocene mammal biostratigraphy of the Valley of Lakes in Mongolia

**DOI:** 10.1007/s12549-016-0264-x

**Published:** 2016-12-15

**Authors:** Mathias Harzhauser, Gudrun Daxner-Höck, Margarita A. Erbajeva, Paloma López-Guerrero, Olivier Maridet, Adriana Oliver, Werner E. Piller, Ursula B. Göhlich, Reinhard Ziegler

**Affiliations:** 1 Natural History Museum Vienna, Burgring 7, 1010 Vienna, Austria; 2 Geological Institute, Siberian Branch, Russian Academy of Sciences, Ulan-Ude; Sahianova Str., 6a, 670047 Ulan-Ude, Russia; 3 Departamento de Paleontología, Facultad de Ciencias Geológicas, Universidad Complutense de Madrid, C/ José Antonio Novais, 2, 28040 Madrid, Spain; 4 Jurassica Museum, Fontenais 21, 2900 Porrentruy, Switzerland; 5 Department of Geosciences, Earth Sciences, University of Fribourg, Chemin du Musée 6, Pérolles, 1700 Fribourg, Switzerland; 6 Paleobiology Department, Museo Nacional de Ciencias Naturales-CSIC, C/ José Gutiérrez Abascal, 2, 28006 Madrid, Spain; 7 Institute of Earth Sciences, NAWI Graz Geocenter, University of Graz, Heinrichstraße 26, 8010 Graz, Austria; 8 Staatliches Museum für Naturkunde Stuttgart, Rosensteinstraße 1, 70191 Stuttgart, Germany

**Keywords:** Oligocene, Miocene, Mongolia, Mammals, Biozones

## Abstract

The Taatsiin Gol Basin in Mongolia is a key area for understanding the evolution and dispersal of Central Asian mammal faunas during the Oligocene and early Miocene. After two decades of intense fieldwork, the area is extraordinarily well sampled and taxonomically well studied, yielding a large dataset of 19,042 specimens from 60 samples. The specimens represent 176 species-level and 99 genus-level taxa comprising 135 small mammal species and 47 large mammals. A detailed lithostratigraphy and new magnetostratigraphic and radiometric datings provide an excellent frame for these biotic data. Therefore, we test and evaluate the informal biozonation scheme that has been traditionally used for biostratigraphic correlations within the basin. Based on the analysis of the huge dataset, a formalised biostratigraphic scheme is proposed. It comprises the *Cricetops dormitor* Taxon Range Zone (Rupelian), subdivided into the *Allosminthus khandae* Taxon Range Subzone and the *Huangomys frequens* Abundance Subzone, the *Amphechinus taatsiingolensis* Abundance Zone (early Chattian), the *Amphechinus major* Taxon Range Zone (late Chattian), subdivided into the *Yindirtemys deflexus* Abundance Subzone and the Upper *Amphechinus major* T. R. Z., and the *Tachyoryctoides kokonorensis* Taxon Range Zone (Aquitanian). In statistical analyses, samples attributed to these biozones form distinct clusters, indicating that each biozone was also characterised by a distinct faunal type.

**Electronic supplementary material:**

The online version of this article (doi:10.1007/s12549-016-0264-x) contains supplementary material, which is available to authorized users.

## Introduction

The Oligocene and Miocene terrestrial deposits of the Valley of Lakes in Mongolia are outstanding regarding the rich and stratigraphically dense successions of mammal assemblages. The semi-desert landscape provides vast outcrops and enables intense sampling. During eight field-campaigns from 1995–2012, our team discovered 26 natural outcrops in the Taatsiin Gol Basin. In total, over 90 samples were collected from the Hsanda Gol and Loh formations (see Daxner-Höck et al. 2017, this issue for details on geological setting, logs and sample positions). The stratigraphic position of the samples is inferred from their relative positions within the sections and corroborated by stratigraphic tie points provided by radiometric dating (Höck et al. 1999) and magnetostratigraphy (Sun and Windley 2015).

In addition, an informal biozonation scheme for Oligocene and Miocene mammal assemblages of the Valley of Lakes was proposed as a biostratigraphic tool (Daxner-Höck et al. 1997). This zonation scheme was subsequently refined by Daxner-Höck (2001) and Daxner-Höck et al. (2010, 2013, 2014). It is based on characteristic assemblages and co-occurrences of taxa and might best be considered as assemblage-zones. They proved to be highly valuable during fieldwork and enabled detecting depositional gaps in the often very uniform lithologies.

The current biozonation for the Oligocene to early Miocene of Daxner-Höck et al. (2017, this issue) distinguishes 6 units: A, B, C, C1, C1-D and D. Zone E was defined for late Miocene assemblages and is not considered herein. The radiometric and magnetostratigraphic dating of the sections by Höck et al. (1999) and Sun and Windley (2015) suggests an early Rupelian age for Zone A (33.9 Ma to ∼31.5 Ma), a late Rupelian age for Zone B (∼31.5 Ma to ∼28.1 Ma), an early Chattian age for Zone C (∼28.1 Ma to ∼25.6 Ma), a mid-Chattian age for Zone C1 (∼25.6 Ma to ∼24.0 Ma), a latest Chattian age for Zone C1-D (∼24.0 Ma to ∼23.0 Ma) and an Aquitanian age for Zone D (∼23.0 Ma to ∼21.0 Ma). The exact boundaries, however, are undefined due to the incomplete sedimentary record and the irregular occurrence of fossil-rich beds.

Herein, we propose a formal definition of the informal biozones including explicit boundaries for each zone based on first and last appearance data of relevant taxa. We evaluate which species are significant and frequent enough to be detected in samples of a certain biozone. These taxa are then chosen to name and define the biozones. The biozones should be defined according to the International Stratigraphic Guide (Hedberg 1976; Salvador 1994; Steininger and Piller 1999; Murphy and Salvador 1999). The first and last records of species and genera could be chosen to define these zones. In some cases, these occurrences might represent First Appearance Datums (FADs) and Last Appearance Datums (LADs) – as far as terrestrial records allow detecting FADs at all. Unfortunately, the central Asian mammal stratigraphy is still too poorly resolved to distinguish between regional and large-scale patterns. We therefore restrict our zonation to the Valley of Lakes and treat the respective occurrences in the individual sections as First Occurrence Datums (FODs) and Last Occurrence Datums (LODs). The assumption is that these are more or less synchronous within the basin. In modification of the original FOD and LOD concept (see above), we adopt the “lowermost occurrence” (LO) and “highest occurrence” (HO) concept applied by many authors to define stratigraphic surfaces instead of single points (e.g. Aubry and Van Couvering 2005; Wade et al. 2011).

For practical reasons, the name-giving taxon of a biozone should be frequent enough to be detected in samples of reasonable size. Accordingly, most of the rare species and genera discussed above should be excluded from biozone definitions due to their spotty occurrence. Steininger and Piller (1999) summarised the requirements for the definition of a biozone as follows:

1. Definition of the biozone type (e.g. Range Zone, Abundance Z., Assemblage Z. etc.).
2. Clear nomenclatorial and taxonomic status of the name-giving taxon, ideally accompanied by an illustration.
3. Description of the type- and reference sections containing the biozone, if appropriate.

To conform to point 2 and 3, we refer to the descriptions and illustrations in the taxonomic monographs treating the Mongolian Oligocene/Miocene mammal faunas, and to comprehensive illustrations of marsupials, eulipotyphlans and rodents in Daxner-Höck et al. (2017, this issue, figs. 32–62). For the type- and reference sections, we refer to the comprehensive description of the sections in Daxner-Höck et al. (2017, this issue).

## Material and methods

Bulk samples of one to several tons were taken from more than 90 fossil-bearing horizons and screened for fossil mammal remains. The samples were then split into systematic groups, identified and quantified by specialists, and published in numerous taxonomic papers (see Daxner-Höck et al. 2017, this issue for full references). We compiled a dataset of 19,042 specimens from 60 samples based on these published occurrence data of Oligocene to early Miocene mammals in the Valley of Lakes (electronic supplement Table 1). The specimens represent 176 species-level and 99 genus-level taxa comprising 135 small and 47 large mammal species. The sampling method clearly focused on small mammals, and larger mammals might therefore be underrepresented, despite their high palaeoecological significance. For each taxon, the number of occurrences was counted per sample. Each specimen was counted as 1; the counts were transferred into percentages (per sample or biozone) and then arcsine-root transformed to balance very high specimen numbers (Linder and Berchtold 1976; Zuschin and Hohenegger 1998). In addition, we transformed this data matrix into a presence/absence matrix.

**Fig. 1:**
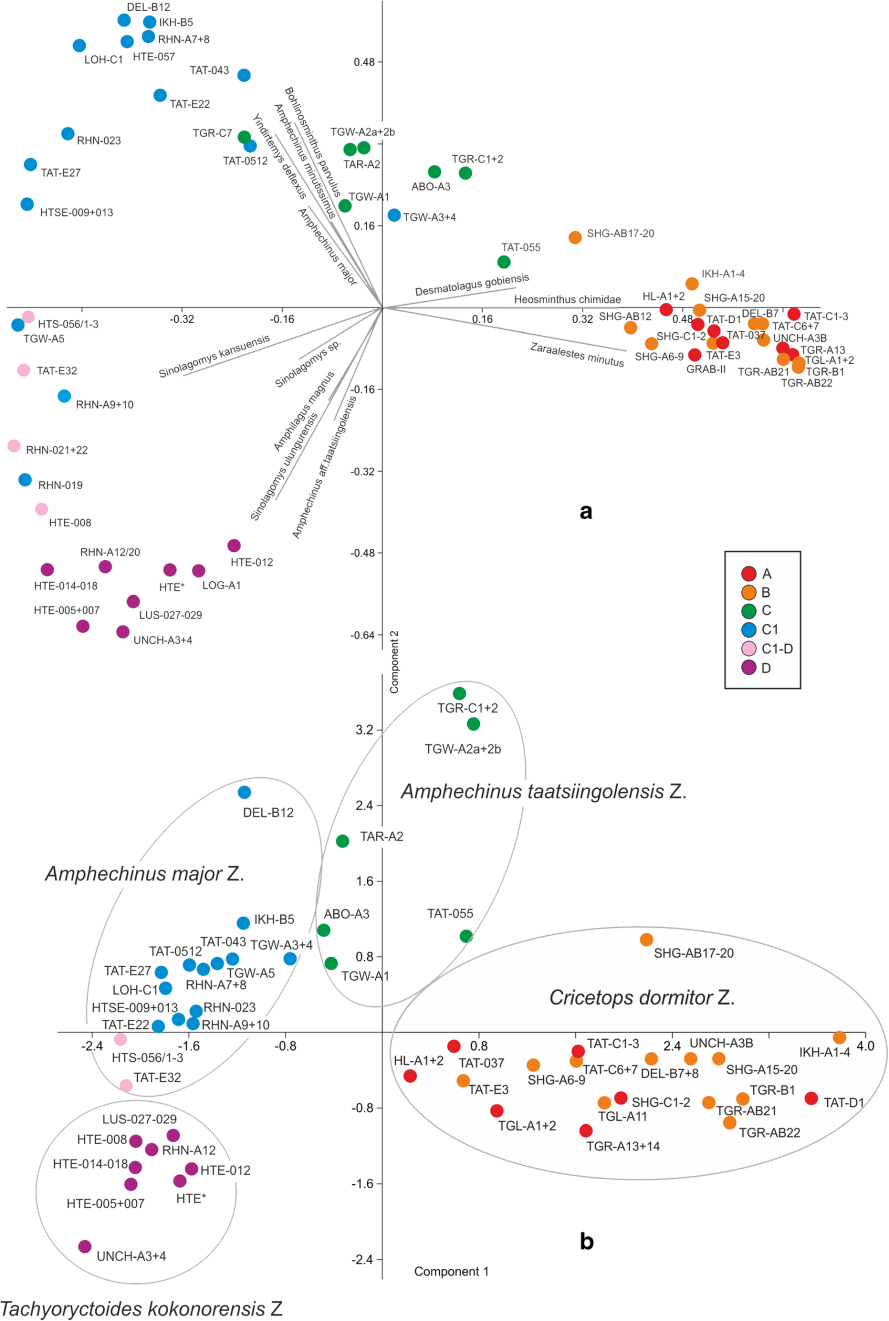
Principal Component Analyses of the Oligocene and Miocene fossil-bearing samples from the Valley of Lakes. *Colour codes* of samples correspond to the assignment to a zone as proposed by Daxner-Höck et al. (2017, this issue). **a** PCA based on percentages of each species per sample (arcsine transformed); species documented by <30 counts in the total dataset were excluded. **b** PCA based on presence/absence matrix; samples with <10 species were excluded; the clusters clearly correspond to the biozones as defined herein

**Fig. 2:**
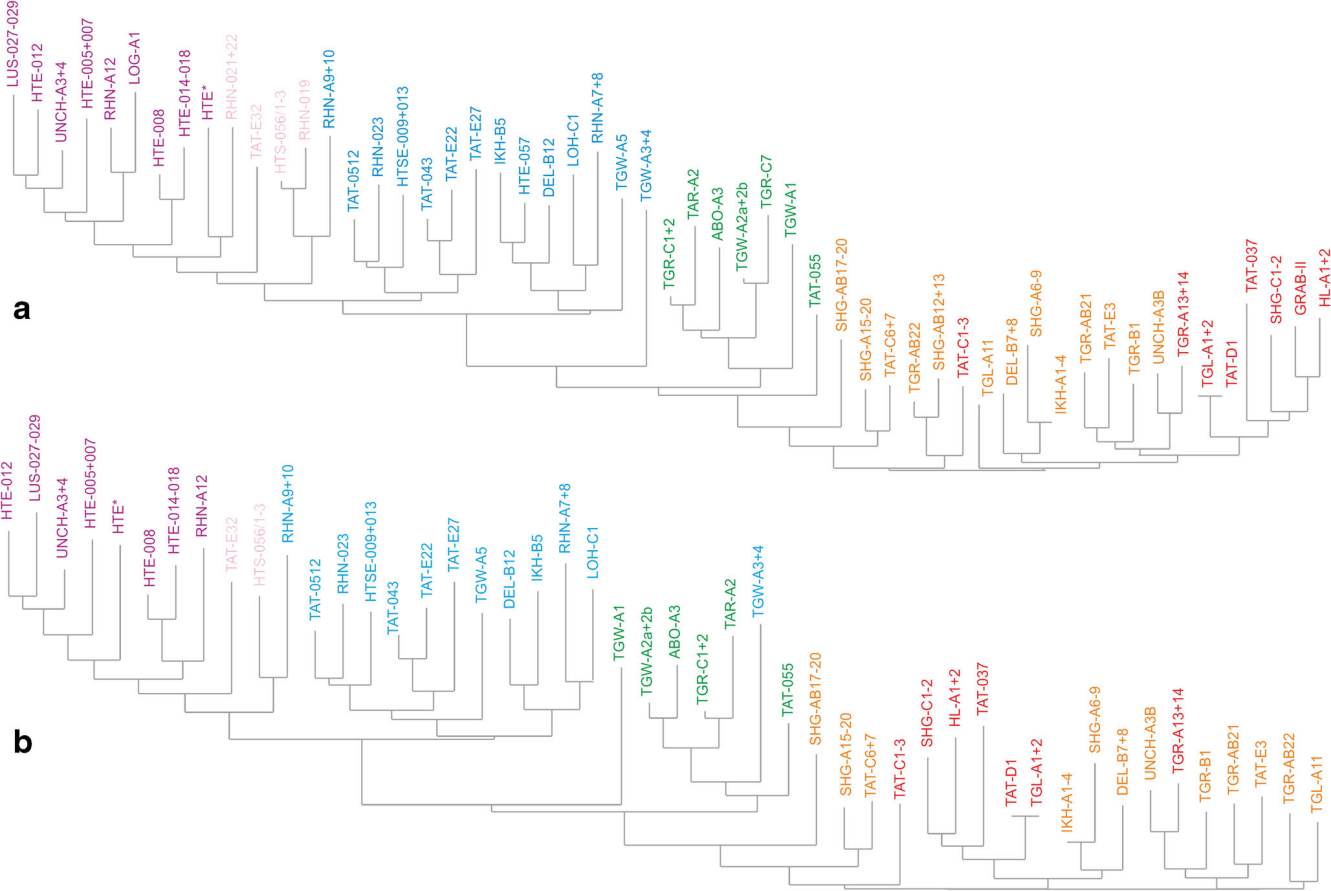
Neighbour-Joining Analyses of the Oligocene and Miocene fossil-bearing samples from the Valley of Lakes (*same colour code* as used in Fig. 1). **a** NJA based on percentages of each species per sample (arcsine transformed); species documented by <30 counts in the total dataset were excluded. **b** NJA based on presence/absence matrix; samples with <10 species were excluded

To detect similarities between samples and sample-groups, we computed a Principal Component Analysis (PCA) (Fig. 1) and a Neighbour-Joining Analysis (NJA, Saitou and Nei 1987) (Fig. 2) for both data sets (counts, presence/absence) using the PAST software-package (Hammer et al. 2001). This method allows defining clusters characterised by the presence and/or abundance of certain taxa. A priori, these clusters do not necessarily correspond to biostratigraphic units; they could also reflect different ecological conditions.

**Fig. 3:**
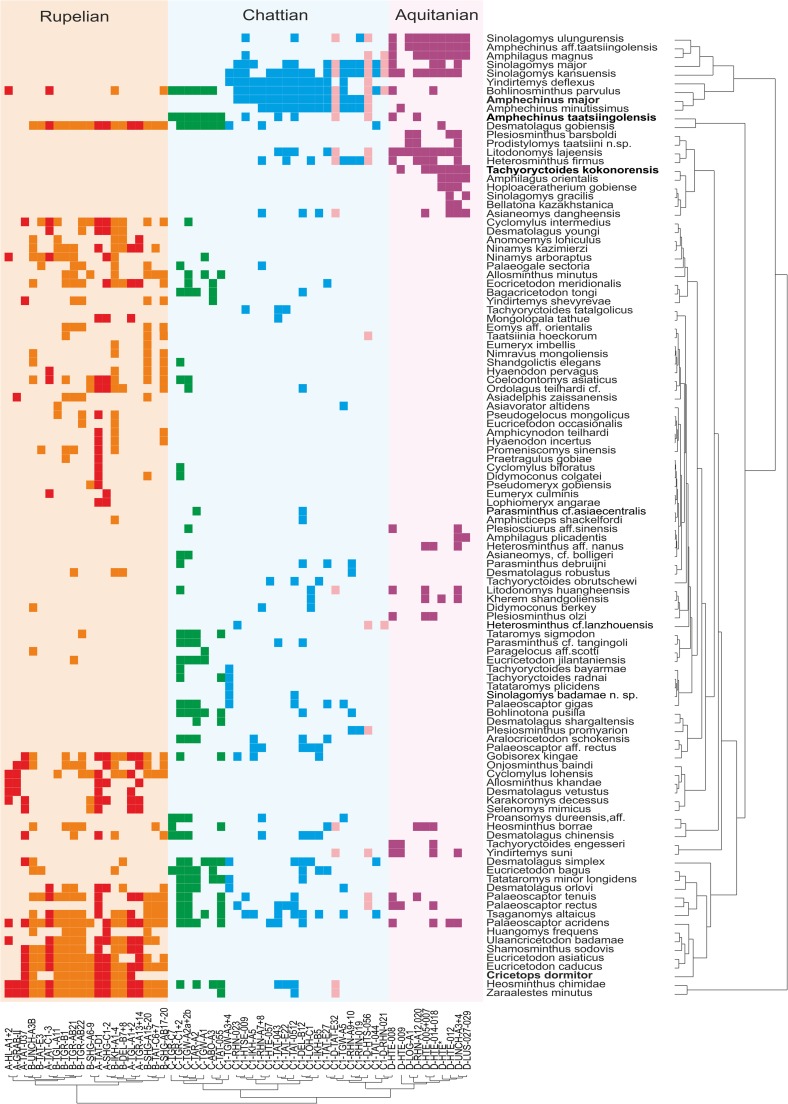
Two-way cluster analysis (Ward’s method) based on counts of species-level taxa; singletons were removed prior to analysis, and only samples with at least 5 species were included (57 samples, 110 species); name-giving species for biozones are printed in *bold*

**Fig. 4:**
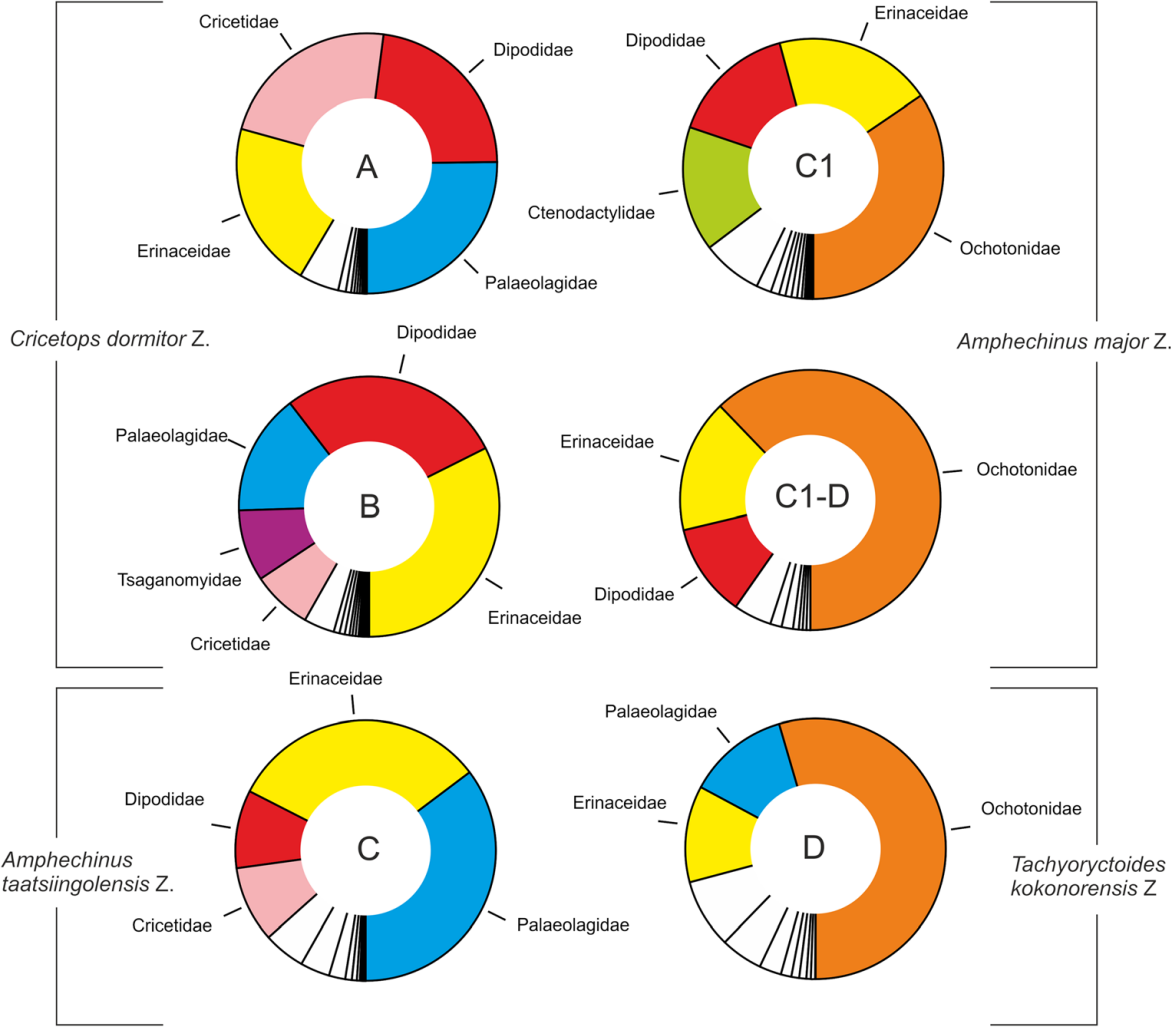
Specimens (counts) per family for each zone; note that Zone C1-D is poorly sampled

Samples containing less than 10 species-level taxa and species, which are represented by less than 30 total counts, were removed prior to analysis to reduce noise by singletons. In addition, we performed coupled Q-mode/R-mode cluster analyses (CA) (Ward’s method) based on abundance data (only samples with at least 5 species were included; singletons were removed; no sp. identifications) (Fig. 3). The full dataset was used to define the biozones (Fig. 4).

All material is stored in the collections of the Natural History Museum Vienna and the Institute of Palaeontology and Geology of the Mongolian Academy of Sciences in Ulaanbaatar.

## Results

The biotic content of the informal biozones based on our compiled dataset can be summarised as follows:

- **Zone A** assemblages (3556 specimens, 8 samples) include 69 species in 43 genera (G/S = 0.62) (electronic supplement Table 1). Overall, rodents are the most common group in our fossil assemblage (Fig. 4), suggesting that they dominated the living mammal communities. At the family level, the samples are dominated by Palaeolagidae (25.2%, La), Dipodidae (22.8%, R), Cricetidae (22.8%, R) and Erinacidae (20.8%, E). The most common species in our assemblage are *Heosminthus chimidae* (19.6%, R), *Zaraalestes minutus* (18.3%, E), *Cricetops dormitor* (15.1%, R), *Desmatolagus gobiensis* (13.6%, La) and *Desmatolagus* sp. 1 (7.5%, La). Fourteen species are unique to Zone A and not recorded in any other zone. These species are *Asiadelphis tjutkovae* (M), *Asiapternodus mackennai* (E), *Cricetops minor* (R), *Allosminthus khandae* (R), *Eomys* cf. *orientalis* (R), *Prosciurus*? *mongoliensis* (R), *Prosciurus*? nov. sp. (R), *Desmatolagus vetustus* (La), *Hyaenodon mongoliensis* (Cr.), *Gobimeryx dubius* (A), *Lophiomeryx angarae* (A), *Lophiomeryx* sp. 1 (A) and *Eumeryx culminis* (A). Combined, they form less than twenty (18.8%) percent of the total number of specimens in the assemblage. In particular, the rare occurrences of the large mammals raise doubts about their biostratigraphic value. *Asiapternodus* (E), *Prosciurus*? (R) and *Lophiomeryx* (A) are restricted to zone A. Given the extreme scarcity of these genera, it is difficult to decide if their presence/absence is a biostratigraphic signal or simply a result of sampling bias.
  We lack a comparable data set for Eocene assemblages of the Valley of Lakes. Therefore, we compare the assemblages with those from the Ergilin Dzo Fm. in southern Mongolia (Dashzeveg 1993). Accordingly, more than 50% of the genera have their First Occurrence Datum (FOD) in Zone A: *Palaeoscaptor* (E), *Gobisorex* (E), *Ordolagus* (La), *Bohlinosminthus* (R), *Coelodontomys* (R), *Cricetops* (R), *Cyclomylus* (R), *Huangomys* (R), *Karakoromys* (R), *Mongolopala* (R), *Ninamys* (R), *Onjosminthus* (R), *Paracricetodon* sp. (R), *Witenia* sp. (R), *Promeniscomys* (R), *Selenomys* (R), *Shamosminthus* (R), *Sinolagomys* (R), *Tsaganomys* (R), *Ulaancricetodon* (R), *Yindirtemys* (R), *Pseudogelocus* (A), *Pseudomeryx* (A), *Amphicynodon* (Ca). Of these, only *Cricetops* (15.1%), *Selenomys* (4.4%) and *Tsaganomys* (3.1%) are present in larger numbers.
- **Zone B** assemblages (5865 specimens, 13 samples) represent 83 species in 52 genera (G/S = 0.63) (electronic supplement Table 1). At the family level, Erinacidae (32.4%, E), Dipodidae (28.0%, R) and Palaeolagidae (15.1%, La) predominate, with lower numbers of Tsaganomyidae (8.9%, R) and Cricetidae (7.4%, R) (Fig. 4). The most common species are *Zaraalestes minutus* (23.9%, E), *Heosminthus chimidae* (23.3%, R), *Desmatolagus gobiensis* (8.5, La), Tsaganomyidae sp. 1. (7.6%, R) and *Palaeoscaptor acridens* (4.6%, E). 12.1% of the species are known only from Zone B: *Zaraalestes* sp. 1 (E), *Eucricetodon occasionalis* (R), *Eomys* aff. *orientalis* (R), *Eomys* sp. (R), *Shamosminthus* sp. (R), *Hyaenodon eminus* (Cr), *Hyaenodon gigas* (Cr), *Nimravus mongoliensis* (Ca), *Ergilictis* sp. 1 (Le) and *Eumeryx imbellis* (A). All these taxa are rarely detected and documented by a few specimens only. Their use for biostratigraphy is therefore very limited. The rare genera *Nimravus* (Ca) and *Ergilictis* (Le) are recorded only from zone B. Eleven genera have their oldest record in Zone B and might represent FODs for the late Rupelian: *Taatsiinia* (E), Crocidosoricinae gen. 1 (E), Heterosoricinae gen. 1 (E), *Tataromys* (R), *Asiavorator* (Ca), *Shandgolictis* (Ca), *Palaeogale* (Ca), *Amphicticeps* (Ca), *Eumeryx* (A), *Prodremotherium* (A), *Paragelocus* (A). Unfortunately, all these genera are very rare – accounting for less than 1.4% of the specimen numbers in the intensively sampled beds of Zone B. Their use for biostratigraphy is thus very limited. Eighteen genera disappear at the boundary of zones B/C. Of these, only *Cricetops* (2.5%, R) and *Ulaancricetodon* (1.7%, R) appear in noteworthy numbers and are candidates to define the B/C boundary based on their highest occurrence (HO). The other genera, mainly rodents, ruminants and carnivores, are very rare.
  Uniting all samples assigned to zones A and B yields 99 species-level taxa. Forty-nine species are restricted to this interval. Of these, *Cricetops dormitor* (7.2%, R) is the most abundant, followed by *Huangomys frequens* (2.0%, R), *Selenomys mimicus* (1.7%, R), *Shamosminthus sodovis* (1.4%, R), *Eucricetodon asiaticus* (1.4%, R), *Ulaancricetodon badamae* (1.3%, R) and *Eucricetodon caducus* (1.0%, R). All other species account for less than 1% each. Similarly, the number of genera restricted to both zones increases distinctly to 17 (*Asiadelphis* (M), *Asiapternodus* (E), *Promeniscomys* (R), *Karakoromys* (R), *Huangomys* (R), *Ardynomys* (R), *Anomoemys* (R), *Onjosminthus* (R), *Ulaancricetodon* (R), *Selenomys* (R), *Cricetops* (R), *Paracricetodon* (R), *Eomys* (R), *Prosciurus?* (R), *Praetragulus* (A), *Miomeryx* (A), *Gobimeryx* (A), *Pseudomeryx* (A), *Pseudogelocus* (A), *Lophiomeryx* (A), *Nimravus* (Ca) and *Ergilictis* (D)).
- *Zone C* assemblages (3587 specimens, 7 samples) represent 67 species-level taxa in 46 genera (G/S = 0.67) (electronic supplement Table 1). At the family level, Palaeolagidae (35.3%, La) and Erinacidae (32.2%, E) are by far the most dominant groups, followed by Dipodidae (9.7%, R) and Cricetidae (9.4%, R) (Fig. 4). *Amphechinus taatsiingolensis* (27.2%, E), *Desmatolagus gobiensis* (17.5%, La), *Bohlinosminthus parvulus* (6.7%, R), *Desmatolagus simplex* (5.4%, La), *Desmatolagus* sp. 1 (5.2%, La) and *Eucricetodon bagus* (5.1%, R) are more abundant; all others account for less than 5% each. 13.6% of the taxa are restricted to zone C: *Exallerix pustulatus* (E), *Asianeomys bolligeri* (R), *Yindirtemys* aff. *ulantatalensis* (R), *Shamosminthus tongi* (R), *Argyromys cicigei* (R), Ansomyinae indet. (R), *Desmatolagus shargaltensis* (La), *Dremotheriu*m cf. *guthi* (A), Bovidae sp. 1 (A). Of these, only *Desmatolagus shargaltensis* accounts for about 1.0% of the total assemblage of Zone C, whereas all others are negligible in numbers.
  The rare *Argyromys* (R), *Dremotherium* (A) and Bovidae gen. 1 (A) are recorded only from C. Seventeen genera have their oldest records in Zone C: *Amphechinus* (E), *Exallerix* (E), *Ansomys* (R), *Aralocricetodon* (R), *Argyromys* (R), *Asianeomys* (R), *Bagacricetodon* (R), *Bohlinotona* (R), *Litodonomys* (R), *Palaeohypsodontus* (A), *Parasminthus* (R), *Plesiosciurus* (R), *Plesiosminthus* (R), *Proansomys* (R), *Tachyoryctoides* (R), *Dremotherium* (A) and Bovidae gen. 1 (A). Of these, only *Amphechinus* occurs in large numbers (27.5%), and its occurrence in Zone C can be reliably classified as a First Occurrence Datum (FOD). For the other taxa, a sampling bias for older samples cannot be fully excluded, although this is unlikely for most of the rodent genera. Exits at the boundary of zones C/C1 are evident for 13 genera, which in total represent only 1% of the specimens from Zone C samples. Thus, although the absence of taxa such as *Allosminthus* (R), *Argyromys* (R) and *Shamosminthus* (R) in younger samples may indeed point to their LODs at the C/C1 boundary, their rare occurrence disqualifies them as useful biostratigraphic markers.
- **Zone C1** assemblages (2295 specimens, 19 samples) represent 78 species-level taxa in 45 genera (G/S = 0.58) (electronic supplement Table 1). The most important family in Zone C1 is the Ochotonidae (34.5%, La) accompanied by fewer Erinacidae (19.6%, E), Dipodidae (15.6%, R) and Ctenodactylidae (15.5%, R) (Fig. 4). The most abundant species are *Sinolagomys kansuensis* (24.6%, La), *Yindirtemys deflexus* (14.2%, R), *Bohlinosminthus parvulus* (11.7%, La) and *Amphechinus major* (10.3%, E); all others account for less than 5% each. 21.8% of the taxa are restricted to Zone C1: *Tavoonya altaica* (E), *Palaeoscaptor* aff. *rectus* (E), *Sinolagomys badamae* (La), *Yindirtemys birgeri* (R), *Plesiosminthus asiaticus* (R), *Tachyoryctoides obrutschewi* (R), *Tachyoryctoides tatalgolicus* (R), *Tachyoryctoides bayarmae* (R), *Eucricetodon* sp. (R), *Didymoconus* sp. (Le), *Amphitragulus* sp. (A), Bovidae gen. 2 (A), *Gobiocerus* sp. (A), *Paraceratherium* sp. (P), *Benaratherium* sp. (P), *Aceratherium pauliacense* (P) and Elasmotheriini gen. 1 (P). None of these species contributes to the assemblages in larger numbers and, combined, they account for <3% of the total counts.
*Tavoonya* (E), *Amphitragulus* (A), Bovidae gen. 2 (A), *Gobiocerus* (A), *Paraceratherium* (P), *Benaratherium* (P), *Aceratherium* (P) and Elasmotheriini gen. 1 (P) are known only from Zone C1. Eleven genera occur in samples of Zone C1 for the first time: *Amphilagus* (La), *Tavoonya* (E), *Heterosminthus* (R), *Kherem* (R), Bovidae gen. 2 (A), *Amphitragulus* (A), *Gobiocerus* (A), Elasmotheriini gen. 1 (P), *Aceratherium* (P), *Benaratherium* (P) and *Paraceratherium* (P). All are very rare and account for less than 2.4% of the specimen numbers of Zone C1 samples. Twenty-six genera of Zone C1 were not detected in younger samples. This high number, however, is at least partly an artefact due to the comparatively poor sampling of zone C1-D. Moreover, all genera are very rare and would be poor candidates to base a biostratigraphic zonation on their potential LOD at the C/C1-D boundary.
- **Zone C1-D:** This is the least sampled zone, with only 423 specimens from 3 samples, which yielded 26 species in 15 genera (G/S = 0.58) (electronic supplement Table 1). Despite the poor sampling, the overall pattern on the family level is comparable to Zone C1, with Ochotonidae (62.2%, La) as the most important group followed by Erinacidae (16.6%, E) and Dipodidae (11.6%, R) (Fig. 4). *Sinolagomys kansuensis* (32.6%, La), *Sinolagomys* sp. 1 (21.5%, La), *Amphechinus taatsiingolensis* (7.1%, E), *Amphechinus major* (5.2%, E) and *Yindirtemys deflexus* (4.3%, R) are the most important constituents in the samples. None of the species and genera are restricted to this zone and no genus displays its FOD.
*Yindirtemys deflexus* (4.3%, R) and *Heterosminthus* (3.6% R), however, are well represented in zone C1-D but unknown from younger samples. Their absence in the Zone D samples thus reliably marks their LODs at the Oligocene/Miocene boundary.
- **Zone D** assemblages (3315 specimens, 10 samples) represent 54 species in 33 genera (G/S = 0.61) (electronic supplement Table 1). At the family level, Ochotonidae (54.5%, La) remain the dominant group accompanied by Palaeolagidae (12.6%, La), Erinacidae (12.0%, E) and Dipodidae (8.6%, R) (Fig. 4). The most important taxa are *Sinolagomys ulungurensis* (28.7%, La), *Sinolagomys* sp. 1 (10.9%, La), *Amphechinus* aff. *taatsiingolensis* (10.1%, E), *Amphilagus magnus* (8.3%, La) and *Sinolagomys kansuensis* (5.1%, La); all other species account for less than 5% each. 44.4% of the assemblage is restricted to this zone (note that this does not exclude occurrence in younger deposits) and includes: *Exallerix* sp. (E), *Amphechinu*s aff. *taatsiingolensis* (E), Pteromyini sp.1 (R), *Eutamias* sp. (R), *Prodistylomys taatsiini* (R), *Prodistylomys mongoliensis* (R), *Prodistylomys* sp. (R), *Yindirtemys suni* (R), *Plesiosminthus olzi* (R), *Plesiosminthus barsboldi* (R), *Heterosminthus* aff. *nanus* (R), *Tachyoryctoides kokonorensis* (R), *Tachyoryctoides engesseri* (R), *Ayakozomys* sp. (R), *Democricetodon sui* (R), *Ansomys* sp. (R), *Primus* sp. (R), *Amphilagus orientalis* (La), *Amphilagus plicadentis* (La), *Bellatona kazakhstanica* (La), *Bellatona yanghuensis* (La), *Alloptox minor* (La), *Amphilagus complicidens* (La), *Sinolagomys gracilis* (La), *Hoploaceratherium gobiense* (P) and *Caementodon* sp. (P). Most of these taxa are very rare; only *Amphechinus* aff. *taatsiingolensis* (10.1%, E), *Tachyoryctoides kokonorensis* (4.2%, R) and *Amphilagus orientalis* (2.8%, La) are comparatively frequently detected in the samples. *Bellatona* (La), *Alloptox* (La), Pteromyini gen. 1 (R), *Eutamias* (R), *Prodistylomys* (R), *Ayakozomys* (R), *Democricetodon* (R), *Primus* (R), *Hoploaceratherium* (P) and *Caementodon* (P) are not known from older zones, and 10 genera display their oldest recorded occurrence within Zone D: *Bellatona* (La), *Alloptox* (La), *Ayakozomys* (R), *Democricetodon* (R), *Eutamias* (R), Pteromyini gen. 1 (R), *Primus* (R), *Prodistylomys* (R), *Caementodon* (P), *Hoploaceratherium* (P). Again, these genera represent only a minor part of the assemblage. The relatively most common ones are *Prodistylomys* (1.3%) and *Bellatona* (0.9%); all others contribute less than 1.3% to the assemblages.

To improve the informal biozone scheme of Höck et al. (1999) and subsequent authors, we checked if samples assigned to a certain zone form distinct clusters in a PCA (Fig. 1), NJA (Fig. 2) and CA (Fig. 3). This approach is based on the assumption that assemblages and samples from a certain biozone are more similar in composition than samples from other biozones. This similarity is thought to reflect a largely identical evolutionary level and comparable large-scale palaeoecological conditions shaping the assemblages within a biozone.

All analyses revealed distinct groupings that correspond well with the informal biozones. Zones A and B are exceptions because they are not well resolved (Fig. 1), although some weak grouping is expressed in the NJA (Fig. 2). In all analyses, however, samples from zones A and B form a very distinct cluster well separated from other samples. In the PCA based on counts, the frequent occurrence of *Heosminthus chimidae* and *Zaraalestes minutus* characterises this A/B cluster. The deep split between this cluster and all other samples is also documented in the cluster analysis (Fig. 3); the simultaneous R-mode clustering is less distinct due to the many species persisting into younger strata, but still shows a compact grouping of taxa including *Heosminthus*, *Zaraalestes*, *Cricetops*, *Eucricetodon*, *Ninamys* and many others.

Samples from Zone C form another distinct cluster, which grades into the well-defined cluster of Zone C1-samples (Fig. 1). Especially in the NJA, these samples cluster between A/B and C1 samples, being overall more similar to C1 (Fig. 2). The frequent occurrence of *Desmatolagus gobiensis* is typical for zone C but does not separate it from Zone B, in which this species is also very frequent.

Samples of Zone C1 also clearly group together in all analyses, with the exception of sample TGW-A3 + 4, which clusters within the Zone C samples in some analyses. This may partly be explained by the few taxa and individuals (12/87) in this sample. The dominant taxa, forcing the grouping of the samples of the C1-cluster in the PCA, are *Yindirtemys deflexus*, *Amphechinus minutissimus* and *Bohlinosminthus parvulus*. The same taxa appear in the respective cluster in the R-mode clustering (Fig. 3). Samples from the rather poorly sampled zone C1-D plot between C1 and D samples in all analyses, but are closer to or even overlap with the C1 cluster. The high contribution by *Sinolagomys kansuensis* is the major factor explaining the grouping in the PCA, without separating it from C1 and D, where this species is also frequent. Samples assigned to Zone D form another very clear cluster in all analyses. The PCA based on specimen counts suggests that *Amphilagus magnus*, *Sinolagomys ulungurensis* and *Amphechinus* aff. *taatsiingolensis* are among the most important constituents. Similarly, taxa from this zone form a distinct group in the R-mode clustering.

In conclusion, the separation between zones A and B is not well resolved in these analyses. Zones C, C1 and D are statistically well supported; Zone C1-D is poorly defined due to the low number of samples. Overall, the arrangement of the Chattian to Aquitanian samples of zones C, C1, C1-D and D suggests a rather continuous development and a distinct separation from the Rupelian samples of zones A and B. Based on these results, we choose typical and frequent taxa to propose the following formal biozones:

- *Cricetops dormitor* Taxon Range Zone
**Type:** Taxon Range Zone, defined by the LO and HO of the rodent species *Cricetops dormitor* Matthew and Granger, 1923. The name-giving species was described and illustrated in detail by Carrasco and Wahlert (1999, figs 1–4) and Daxner-Höck et al. (2017, this issue, fig. 55/a–e). The *C. dormitor* zone is further characterised by the frequent occurrence of the rodent *Heosminthus chimidae* and the hedgehog *Zaraalestes minutus* as well as the taxon ranges of the rodents *Huangomys frequens*, *Selenomys mimicus*, *Shamosminthus sodovis*, *Eucricetodon asiaticus*, *Ulaancricetodon badama*e and *Eucricetodon caducus*.
  Age and sections: Rupelian; the oldest samples attributed to this biozone come from Hsanda Gol deposits overlying fluvio-lacustrine deposits of the Tsagan Ovo Fm. and underlying basalt I (e.g. Taatsiin Gol, TGR-AB section). These strata are correlated with Chron C12r and the upper part of Chron C13 (see Daxner-Höck et al. 2017, this issue). The youngest samples containing assemblages of this biozone (e.g. Hsanda Gol, SHG-A section) are older than basalt II and Chron C9r, resulting in an upper boundary of 27.4 Ma maximum. Because samples below basalt II (Abzag Ovo section, sample ABO-A3) already belong to the next biozone, the upper boundary of the *Cricetops dormitor* T. R. Z. has to be somewhat older. This boundary might coincide with the Rupelian/Chattian boundary.
**Correlation:** corresponds to zones A and B of Höck et al. (1999).
**Subdivision:** the statistical analyses of the samples of the *Cricetops dormitor* T. R. Z. did not yield clearly separated groups. Nevertheless, a weak grouping is evident in the NJA and some taxa clearly allow distinguishing a lower and an upper part of the biozone, which are defined herein as sub-biozones:
  - *Allosminthus khandae* Taxon Range Subzone
**Type:** Taxon Range Subzone, defined by the LO and HO of the rodent *Allosminthus khandae* (Daxner-Höck 2001). The name-giving species was described and illustrated in Daxner-Höck et al. (2014: 138, fig. 4, Daxner-Höck et al. 2017, this issue, fig. 54/a–e.). In addition, the ranges of *Cricetops minor*, *Prosciurus*? *mongoliensis* and *Desmatolagus vetustus* characterise this subzone. The rare occurrence of these taxa makes detecting this biozone difficult when sample size is small.
**Age and sections:** early Rupelian. The base is defined by the base of the *Cricetops dormitor* Zone; the top coincides with basalt I and lies within Chron C12r, suggesting an absolute age of c. 31.5 Ma. Typical samples of this biozone are found at Taatsiin Gol (TGR-AB section) (Daxner-Höck et al. 2017, this issue).
**Correlation:** corresponds to Zone A of Höck et al. (1999).
  - *Huangomys frequens* Abundance Subzone
**Type:** Abundance Subzone, defined by the frequent occurrence of the rodent *Huangomys frequens* Schmidt-Kittler, Vianey-Liaud and Marivaux, 2007, which was described and illustrated by Schmidt-Kittler et al. (2007): 201, fig. 96 and in Daxner-Höck et al. 2017, this issue, fig. 45/j–p). This species accounts only for <0.03% of the assemblage of the older *Allosminthus khandae* Subzone but rises to 3% in the *Huangomys frequens* Subzone. This subzone is further characterised by the range of *Eucricetodon occasionalis*.
**Age and sections:** late Rupelian; the base coincides with the top of basalt I; the top is defined by the top of the *Cricetops dormitor* Zone; typical outcrops spanning this subzone are exposed at Hsanda Gol in the SHG-A section (Daxner-Höck et al. 2017, this issue).
**Correlation:** corresponds to Zone B of Höck et al. (1999).
- *Amphechinus taatsiingolensis* Abundance Zone
**Type:** Abundance Zone, defined by the lowest occurrence (LO) and very frequent occurrence of the eulipotyphlan species *Amphechinus taatsiingolensis* Ziegler, Dahlmann and Storch, 2007, which was described and illustrated by Schmidt-Kittler et al. (2007): 96, fig. 11, and Daxner-Höck et al. 2017, this issue, fig. 34/a–n). This species accounts for about 27% of the samples in this biozone but represents <0.5% of the samples in the subsequent biozone. Both biozones are well sampled, suggesting that this abundance pattern represents local conditions during deposition of the fossils. This biozone is also characterised by the FOD and frequent occurrence of *Tataromys minor longidens* and the frequent occurrence of *Eucricetodon bagus* and *Desmatolagus simplex.* It is further distinguished by incorporating the complete temporal range of *Desmatolagus shargaltensis.*
**Age and sections:** early Chattian; the base is defined by the oldest samples of the biozone below basalt II and a position within Chron C9r (Daxner-Höck et al. 2017, this issue), suggesting an age of about 27.6 Ma. The base of this biozone corresponds roughly with the base of the Chattian. The top of the biozone falls within Chron C8n.2n, ranging around 25.6 Ma (Daxner-Höck et al. 2017, this issue). A typical section bearing assemblages of the *Amphechinus taatsiingolensis* A. Z. is section TGR-C at Taatsiin Gol (Daxner-Höck et al. 2017, this issue).
**Correlation:** corresponds to Zone C of Höck et al. (1999).
**Subdivision:** none.
- *Amphechinus major* Taxon Range Zone
**Type:** Taxon Range Zone, defined by the LO and HO of the eulipotyphlan species *Amphechinus major* Ziegler, Dahlmann and Storch, 2007, which was described and illustrated by Ziegler et al. (2007: 106, fig. 13) and Daxner-Höck et al. (2017, this issue: fig. 35/j–q). This biozone is characterised by the total ranges of *Yindirtemys deflexus* and *Plesiosminthus promyarion* and the FODs of the genera *Amphilagus*, *Tavoonya* and *Heterosminthus.*
**Age and sections:** late Chattian; the base of the biozone falls within Chron C8n.2n, ranging around 25.6 Ma (Daxner-Höck et al. 2017, this issue). No radiometric and palaeomagnetic dates are available for the upper part of the biozone, which is above Chron C7n.2n and below lower Miocene deposits.
  Correlation: corresponds to zones C1 and C1-D of Höck et al. (1999).
**Subdivision:** the *Amphechinus major* T. R. Z. is divided into a longer lower unit and a shorter but less sampled upper unit. The lower unit is defined herein as a sub-biozone:
  - *Yindirtemys deflexus* Abundance Subzone
**Type:** Abundance Zone, defined by the FOD and very frequent occurrence of the rodent *Yindirtemys deflexus* (Teilhard de Chardin, 1926), which was described and illustrated by Schmidt-Kittler et al. (2007: 191, figs 49–93), Oliver and Daxner-Höck (in press): X, figs 2–3), and in Daxner-Höck et al. (2017, this issue, fig. 46/e–k). Although *Yindirtemys deflexus* is only occasionally found in the lowermost part of the upper part of the *Amphechinus major* T. R. Z., *Yindirtemys deflexus* is abundant only in the lower part of the *Amphechinus major* T. R. Z. In addition, this subzone is characterised by frequent occurrences of *Sinolagomys kansuensis*, *Bohlinosminthus parvulus* and *Amphechinus major*.
**Age and sections:** early late Chattian; the base of the *Yindirtemys deflexus* Abundance Subzone is defined by the base of the *Amphechinus major* T. R. Z.; the top falls within Chron C7n.2n (Daxner-Höck et al. 2017, this issue), limiting the upper boundary to about 24.1 Ma. A typical section covering this biozone is the TGR-C section at Taatsiin Gol (Daxner-Höck et al. 2017, this issue).
**Correlation:** corresponds to zone C1 of Höck et al. (1999).
  - Upper *Amphechinus major* T. R. Z.
    The upper *Amphechinus major* zone is dominated by *Sinolagomys* and characterised by generally low diversities of other taxa. *Plesiosminthus promyarion* is more abundant than in the *Yindirtemys deflexus* A. Z., but this may be an artefact of poor sampling. Therefore, we refrain from defining a formal bio-subzone for this interval.
**Age and sections:** latest Chattian; its base lies within Chron C7n.2n (Daxner-Höck et al. 2017, this issue); the top probably correlates with the Oligocene/Miocene boundary, but palaeomagnetic and radiometric dates are missing; typical sections covering this interval are exposed at Huch Teeg (RHN-A section) and at Tatal Gol (TAT-E section).
**Correlation:** corresponds to Zone C1-D of Daxner-Höck et al. (2014).
- *Tachyoryctoides kokonorensis* Taxon Range Zone
**Type:** Taxon Range Zone, defined by the LO and HO of the rodent *Tachyoryctoides kokonorensis* Li and Qiu, 1980, which was described and illustrated in detail by Daxner-Höck et al. (2015: 178, figs 5–6) and Daxner-Höck et al. (2017, this issue, fig. 62/a–e). This biozone is further characterised by abundant *Sinolagomys ulungurensis* and *Yindirtemys suni*, as well as by the total range of *Amphechinus* aff. *taatsiingolensis* and the FODs of the genera *Prodistylomys* and *Bellatona.*
**Age:** Aquitanian; the base coincides with the base of the Miocene part of the Hotuliin Teeg section (sample HTE-009). The top is undefined; the uppermost deposits containing samples of the biozone are exposed at Hotuliin Teeg (HTE section) and Unkheltseg (UNCH-A section) and represented by the so-called Rhino-Sands of Daxner-Höck et al. (2017, this issue). The correlation with the Aquitanian is based on similarities with assemblages from Dzungaria in China (Meng et al. 2006, 2013). No magnetostratigraphic or radiometric dating is available for the sections containing this biozone. Therefore, the proposed correlation is preliminary.
**Correlation:** corresponds to Zone D of Höck et al. (1999).
  Subdivision: none.

## Discussion

In all statistical analyses, the grouping of the samples follows their assignment to biozones. This documents that each biozone is characterised by a distinct faunal type, reflecting a more or less uniform evolutionary level of the various taxa and comparable ecological conditions. Based on the results of the PCA and NJA, we identify a major split between Rupelian faunas of the *Cricetops dormitor* Zone and those of the subsequent Chattian *Amphechinus taatsiingolensis* and *Amphechinus major* zones. The position of the Chattian samples in the scatter plots (Fig. 1) indicates a gradual development of these biozones. The samples of the Aquitanian *Tachyoryctoides kokonorensis* Zone follow this overall (stratigraphic) trend but are more separated, indicating another turnover at the Oligocene/Miocene boundary. These punctuations are most probably the result of climate forcing and corresponding changes in palaeoenvironments (Harzhauser et al. 2016). A detailed reconstruction of the palaeoenvironments is beyond the scope of this paper, but some general conclusions can be drawn:

- Rupelian (*Cricetops dormitor* Taxon Range Zone): the high diversities and similar contributions by Palaeolagidae, Dipodidae, Cricetidae and Erinacidae (Fig. 4) suggest diverse habitats with numerous ecological niches. Most small mammals were ground dwellers, partly adapted to a fossorial lifestyle (e.g. Tsaganomyidae, Wessels et al. 2014). Wonderful discoveries of partly articulated skeletons in fossil burrows provide a particularly poignant example (Daxner-Höck et al. 2017, this issue). Large Cricetidae, such as *Eucricetodon asiaticus* and *E. caducus*, are common. The teeth of these species have brachydont/bunodont crowns, oblique/blunt cusps, a simple occlusal pattern and low crown heights, indicating a diet with an omnivorous component (Williams and Kay 2001; Samuels 2009). Similarly, dental microwear analysis of *E. asiaticus* from Ulantatal (Gomes Rodrigues et al. 2012) indicates that its diet included a mixture of fruits and grasses with a component of animal matter. This implies that patches of forests were present, which is also supported by the rare occurrence of Didelphidae. Although underrepresented in specimen numbers, the high number of Artiodactyla species indicates a rich food supply, which in turn gave rise to a comparably large number of Carnivora and Creodonta. Ephemeral water bodies are indicated by the herpetofauna, particularly by pelobatid frogs, which prefer open landscapes and are adapted to dry habitats (Böhme 2007). During the late Rupelian, changes in palaeoenvironments are reflected in the bio-subzonation. Although the overall diversity of Cricetidae increases, their body size decreases; the dental microwear analysis of *Eucricetodon jilantaiensis* from Ulantatal indicates a diet without fruit and increased consumption of abrasive and fibrous plants (Gomes Rodrigues et al. 2012). The complexity of the occlusal surface of the teeth of cricetids (with several folds on the occlusal surface) point to a strong herbivorous component (Evans et al. 2007; Samuels 2009). Hence, open landscapes became more abundant during the late Rupelian. The Rupelian palaeoenvironment of the Valley of Lakes was probably comparable to the modern Serengeti, with predominately open landscapes.
- Early Chattian (*Amphechinus taatsiingolensis* Abundance Zone): Following the extinction of at least 18 genera near the Rupelian/Chattian boundary, the mammal communities include fewer taxa, and a small number of species dominate the assemblages. Small mammal groups were predominantly ground dwelling and many were probably fossorial (Tsaganomyidae, *Tachyoryctoides*). Forest dwellers were absent and the diversity of large mammals decreased drastically. The dominant Palaeolagidae, with rooted, low crowned teeth, clearly indicate the presence of meadows. Other groups, however, show a tendency towards hypsodonty, lophodonty and/or thick enamel (e.g. *Eucricetodon bagus*, *Yindirtemys ulantatalensis*, *Tachyoryctoides radnai*, *Tataromys plicidens*, *Aralocricetodon*, *Bagacricetodon*, *Argyromys*). *Aralocricetodon* and *Argyromys* are characterised by broad upper molars with straight lamellae. These morphologies imply a highly abrasive diet (Casanovas-Vilar et al. 2011; Gomes Rodrigues et al. 2014). Overall, our data suggest increasing aridification, loss of soft plants and opening of environments.
- Late Chattian (*Amphechinus major* Taxon Range Zone): as in the preceding *Amphechinus taatsiingolensis* Abundance Zone, the small mammals are all ground dwellers and fossorial species are still frequent; arboreal species are completely missing. The rise and dominance of Ochotonidae is the main feature of this biozone, replacing the Rupelian to early Chattian Palaeolagidae. Although the earliest *Sinolagomys* had rooted teeth, all species lack tooth-roots completely. Loss of tooth roots in *Sinolagomys* spp. indicates adaptation to feeding on grass, likely an adaptation to steppe landscapes. The tendency towards rootless teeth and increasing hypsodonty and/or lophodonty in many other small mammal groups (e.g. Ctenodactylidae) is consistent with ongoing climate deterioration within a semi-arid steppe environment.
- Aquitanian (*Tachyoryctoides kokonorensis* Taxon Range Zone): this biozone is characterised by continued dominance of Ochotonidae. Many mammal groups are fully hypsodont or lophodont (e.g. Tachyoryctoididae, Ctenodactylidae) – adaptations to a hard and nutrient-poor food supply associated with an open landscape. Dry but vegetated environments are also indicated by the terrestrial mollusc fauna (Neubauer et al. 2013). The rare occurrence of flying squirrels (Pteromyini) demonstrates some trees because gliding squirrels are strictly arboreal and shun open landscapes (Lu et al. 2013). The sedimentary record, with channels and fluvial gravel, suggests episodic phases of high precipitation, which might have allowed deep-rooting trees to cope with the overall semi-arid climate.

## Conclusion

The statistical analyses of the mammal assemblages clearly support large parts of the informal zonation as used by Höck et al. (1999) and subsequent authors. Here, we created a formal biozonation for the Taatsiin Gol Basin. Our scheme improves the previous informal scheme by focusing on frequently occurring species to define the biozones. Our new formal scheme works excellently within the entire Taatsin Gol Basin. In particular, our new formal scheme increases our ability to recognise subtle differences in the region within formally described time periods. Furthermore, it enables using the faunal composition of a sample to identify its position within the temporal sequence in the region – a particularly useful tool during field work. Moreover, our new biozone scheme minimises sampling bias, which might mask the occurrences and ranges of rare species. Finally, the analysis of the faunal composition for each biozone reveals distinct patterns, with certain taxa dominating the spectra. This faunistic “fingerprint” might allow a much clearer correlation between Oligocene and Miocene mammal faunas across Asia, for which quantitative data and statistical analyses are usually missing.

## Electronic supplementary material

Below is the link to the electronic supplementary material.

ESM 1 Species-level taxa from the Oligocene and lower Miocene deposits of the Taatsiin Gol Basin with specimen counts per sample. Abbreviations: Ma: Marsupialia, R: Rodentia, La: Lagomorpha, E: Eulipotyphla, Le: Leptictida, Cr: Creodonta, Ca: Carnivora, A: Artiodactyla, P: Perissodactyla (XLSX 88.1 kb)
